# Integrated fertilization with organic manure and *Trichoderma* enhances wheat productivity and soil nutrient availability

**DOI:** 10.3389/fpls.2025.1687216

**Published:** 2025-10-14

**Authors:** Li Ren, Jilong Lv, Fuli Zhang, Bingqian Dou, Lantao Li, Yilun Wang, Yinjie Zhang

**Affiliations:** ^1^ College of Resources and Environment, Henan Agricultural University, Zhengzhou, China; ^2^ Management Committee of Henan Zhoukou National Agricultural Science and Technology Park, Zhoukou, Henan, China; ^3^ Henan Province Key Laboratory of Efficient Crop Production and Food Quality Safety, Zhoukou Normal University, Zhoukou, Henan, China

**Keywords:** wheat, organic fertilizer, *Trichoderma*, organic nitrogen fractions, phosphorus form

## Abstract

Reducing chemical fertilizer application combined with organic fertilizers has been demonstrated to be an effective strategy for enhancing soil fertility and increasing crop yields. However, the effects of organic fertilizer combined with *Trichoderma* application on wheat yields and soil nutrients remain poorly understood. Here, a field experiment was conducted in 2022, including: no nitrogen (N) application (NoN), 100% chemical fertilizer N (CN), 80% chemical fertilizer N and 20% manure N (CNM), and 80% chemical fertilizer N, 20% organic fertilizer N, and *Trichoderma* (CNMT) treatments. In comparison to CN, both CNM and CNMT significantly enhanced wheat yield, soil organic matter content, and soil nutrient levels, with CNMT demonstrating a more pronounced effect. CNMT significantly increased wheat yield, grain number, dry matter accumulation in grains and roots by 11.4%, 9.9%, 17.4%, and 11.9%, N accumulation in grains and roots by 24.9%, and 9.83%, and phosphorus (P) accumulation in straw, grains and roots by 13.8%, 33.5%, and 8.9%. CNMT significantly increased the contents of organic matter, hydrolyzable amino sugar N, H_2_O-P, NaHCO_3_-Pi, NaHCO_3_-Po, NaOH-Pi, and NaOH-Po in rhizosphere and non-rhizosphere soils. Redundancy analysis showed that organic matter was the main factor affecting the morphological distribution of N and P in both rhizosphere and non-rhizosphere soils. CNMT significantly increased the diversity of microbial communities and enhanced complexity and stability of the microbial network. Overall, the combination of chemical fertilizer, manure, and *Trichoderma* (80% chemical fertilizer N, 20% organic fertilizer N, and *Trichoderma*) can significantly increase soil organic matter, enhance the potential for N supply, reduce the fixation of P, promote the diversity of soil microbial communities, improve the uptake and utilization of nutrients by crops, and increase wheat yield.

## 1 Introduction

Wheat (*Triticum aestivum L.*) is a key global staple, providing about 20% of the world’s calories and protein (Zhao et al., 2021). Overusing chemical fertilizers in wheat production disrupts soil balance, leading to acidification, compaction, and reduced microbial activity, which ultimately affects wheat yield (Zhou et al., 2019; Yang et al., 2025). Applying organic fertilizers boosts soil fertility and quality, offering a sustainable way to maintain high crop yields and meet the growing global food demand (Zhang et al., 2021; Yin and Cui, 2024). Combining chemical and organic fertilizers is crucial for boosting wheat production, maintaining soil fertility, and supporting sustainable agriculture. However, the effects of using chemical and organic fertilizers along with *Trichoderma* on soil nutrient transformation and wheat yield are still not well understood.

Nitrogen (N) and phosphorus (P) are vital macronutrients for plants, playing a crucial role in the growth and productivity of crops (Sun et al., 2017; Chen et al., 2020b). Agricultural management significantly influences soil N and P availability by regulating the distribution and composition of their organic and inorganic fractions (Bi et al., 2020; Niu et al., 2023). For example, partial substitution of chemical fertilizer with organic fertilizer could accumulate active fractions of the soil organic N pool, enhance soil N supply, and increase mineral N retention in acidic rice fields (Hou et al., 2022). Long-term application of manure facilitates the formation of newberyite, which enhances soil inorganic P availability, and improves the ratio of orthophosphate diesters to monoesters, thereby contributing to the lability of soil organic P in maize-wheat-cotton rotation systems (Liu et al., 2020). Therefore, accurate discrimination and quantification of diverse soil N and P forms are vital for assessing soil N and P availability and transformation characteristics, as well as crucial for optimizing fertilization practices and developing sustainable fertilization management strategies. Moreover, agricultural fertilization management alters soil structure and chemistry, affecting microbial habitats and diversity, which in turn influence biogeochemical cycles (Hartmann and Six, 2023). Soil microorganisms serve as primary agents in the transformation of soil N and P fractions and are crucial for regulating the decomposition of soil organic matter and nutrient cycling (Dai et al., 2019; Chen et al., 2021). Many studies have shown that the application of organic fertilizers can enhance the diversity and functional potential of soil microorganisms (Luo et al., 2019; Yu et al., 2024). The combination of chemical fertilizer and manure increased *phoD* gene abundance and P availability (Estrada-Bonilla et al., 2021), as well as the potential N fixation rate (Dai et al., 2021). It is unknown how the combined application of chemical fertilizer, organic fertilizer, and *Trichoderma* regulates microbial communities in the rhizosphere and non-rhizosphere soils in winter wheat fields.

As a dominant biocontrol strain, *Trichoderma* fungi are highly effective at preventing diseases that affect soil, particularly root rot caused by *Fusarium* spp., *Rhizoctonia* spp., and *Pythium* spp (Gajera et al., 2013). *Trichoderma* enhances root growth and organic secretion, excels in soil nutrient mobilization and absorption, and efficiently improves soil structure and crop growth. For example, colonizing roots with *Trichoderma* can boost nutrient uptake from the soil, significantly improve N use efficiency in crops, and enhance plant health by activating N signaling pathways (Singh et al., 2019). The *Trichoderma* genus can solubilize phosphate and make it accessible to plants by releasing organic acids, enzymes, and phosphatases, which solubilize inorganic P and mineralize organic P (Li et al., 2015b). Research showed that the application of *Trichoderma viride* biofertilizer significantly altered the structure and composition of the microbial community, increased bacterial diversity, and promoted the absorption of fertilizer N by sweet sorghum, consequently improving the efficiency of fertilizer utilization (Wang et al., 2018). Recently, compared with the combined application of cattle manure and green manure, combining cattle manure with *Trichoderma* and green manure application can significantly improve the growth parameters and yield of maize, which indicates that the combined application of manure and *Trichoderma* can enhance fertilizer efficiency (Nascimento et al., 2025). Currently, the organic fertilizer application is a highly recommended fertilization practice, which can improve microbial structure and function, enhance soil nutrient cycling, and boost crop yields (Wei et al., 2021; Yi et al., 2021; Zhang et al., 2025). The co-application of organic fertilizer and *Trichoderma* has shown benefits in crops like maize; the underlying mechanisms are likely crop-specific. The wheat rhizosphere environment, characterized by its fine root system and distinct exudate profile, may host a unique interaction with *Trichoderma*. However, how the combination of Trichoderma with organic amendments alters microbial communities, influences soil nutrient transformation, and impacts crop yield and nutrient uptake is largely unknown.

In this study, a 3-years (2022−2024) wheat-maize field cropping experiment was conducted to investigate the effects of various fertilization strategies, including chemical PK fertilizer, chemical NPK fertilizer, chemical fertilizer plus manure, and chemical fertilizer with manure and *Trichoderma*, on wheat yield and nutrient transformation characteristics. The aims were to i) elucidate the effects of combined manure and *Trichoderma* addition on wheat yield and nutrient uptake, ii) characterize N and P dynamics in rhizosphere and non-rhizosphere soils, and iii) examine the microbial community structure and its relationship with nutrient contents. Understanding these underlying mechanisms provides novel insights for fertilization management in wheat production.

## 2 Materials and methods

### 2.1 Experimental site and design

The field trial started in October 2022 at the Zhoukou Agricultural High-tech Industry Demonstration Zone (114°38′E, 33°37′N), Henan, China. The experimental site is characterized by a warm-temperate continental monsoon climate, with an average annual temperature of 14.6°C and annual precipitation of 790 mm. The test soil is classified as lime concretion black soil, exhibiting the following physicochemical properties: pH 8.2, soil organic matter 15.4 ± 1.2 g kg^−1^, alkali-hydrolyzable N 48.9 mg kg^−1^, ammonium N (NH_4_^+^-N) 4.9 mg kg^−1^, nitrate N (NO_3_^−^-N) 41.2 mg kg^−1^, available P 5.9 mg kg^−1^, and available potassium 176 mg kg^−1^.

Four treatments were used: no N fertilizer (NoN), 100% chemical N fertilizer (CN), 80% chemical fertilizer N + 20% manure N (CMN), and 80% chemical fertilizer N + 20% manure N+*Trichoderma* (CNMT). Three replicates per treatment were carried out in randomized blocks, with plot sizes of 30 m^2^ (3  m × 10  m). Inorganic fertilizers were applied as urea (46%, N), calcium superphosphate (12%, P_2_O_5_) and potassium chloride (60%, K_2_O). Organic fertilizer was applied as pig manure. The same amounts of N, P_2_O_5_, and K_2_O were 225, 150, and 75 kg ha^–1^, respectively. Organic fertilizer and chemical fertilizer were applied as basal fertilizer, which was incorporated into the soil by rotary tillage after broadcasting (at a depth of 10–15 cm). *Trichoderma asperellum* (isolated from wheat rhizosphere in field site) diluted with water at a 1:2 ratio, applied at 2.5 L m^−2^ containing ~10^9^ CFU mL^−1^ by root drenching during the seedling stage, jointing stage (mid-March), and flowering stage (mid-April), ensuring thorough drenching to saturate the root zone. The application rate of 2.5 L m^−2^ for the *Trichoderma* was selected based on preliminary trials which identified this rate as optimal for enhancing plant growth.

### 2.2 Sampling and analysis

At the second wheat maturity period (June 2024), representative 1 m^2^ quadrat plants were collected from each plot. All wheat plants were cut at the crown, and the number of spikes per unit area was counted. After air-drying, the wheat was threshed and weighed, and the yield per unit area was finally calculated. From each plot, 20 representative plants were randomly selected and brought back to the laboratory for the determination of spike number per plant, grain number per spike, and thousand-kernel weight. The wheat roots, straw, and grains were sampled, killed at 105°C for 30 minutes, dried to constant weight at 65°C, weighed, and ground. After digestion with H_2_SO_4_-H_2_O_2_, the total N and P contents in each part of the wheat were determined using an AA3 flow analyzer.

At the same time, rhizosphere soil was collected using the “root-shaking” method. Loose soil was shaken off first, and then soil within 0−1 mm of the root was collected with a sterile brush. The remaining soil was regarded as non-rhizosphere soil. All fresh soil samples were sieved through a 20-mesh sieve, homogenized, and then divided into three portions using the quartering method: one portion of the fresh soil was stored at 4 °C for the determination of NH_4_^+^-N and NO_3_^–^N; another portion was stored at -80 °C for the analysis of soil microbial indicators; and the air-dried soil was sieved through 100-mesh and 20-mesh sieves for the determination of soil physicochemical properties, organic N, and P fractionations. The dichromate (K_2_Cr_2_O_7_) redox titration method was used to measure total organic matter (Mebius, 1960). Mineral N (NH_4_^+^-N and NO_3_^−^-N) were analyzed using a flow injection autoanalyzer (AA3, SEAL, Germany) following extraction with 2 mol L^−1^ KCl. Soil available P was extracted using the Olsen method with 0.5 mol L^−1^ NaHCO_3_. Available potassium (AK) was determined via ammonium acetate extraction and flame photometry.

### 2.3 Soil N and P fractions analysis

Soil organic N fractions were determined by acid hydrolysis (Bremner, 1949; Kowalenko and Babuin, 2009). A 10 g air-dried soil sample (< 0.15 mm) was hydrolyzed with 10 mL of 6 M HCl at 120 °C for 12 h. Organic N was partitioned into acid-hydrolyzable nitrogen (AHN) and acid-insoluble N. AHN was determined by Kjeldahl digestion after subtraction of NH_4_^+^-N. Acid-hydrolyzable ammonium N (AHAN) was quantified via MgO distillation with background NH_4_^+^-N correction. Acid-hydrolyzable amino acid N (AAN) was measured after NaOH hydrolysis and ninhydrin derivatization. Acid-hydrolyzable amino sugar N (ASN) was calculated by subtracting the sum of AHAN and NH_4_^+^-N from the value obtained using phosphate-borate buffer (pH 11.2). The unidentifiable hydrolyzable N (AHUN) fraction was derived by subtracting AHAN, AAN, and ASN from total AHN.

The chemical P fractions were determined via the modified Hedley method (Hedley et al., 1982; Tiessen and Moir, 1993; Sui et al., 1999). Soil samples underwent sequential extraction with deionized water, 0.5 M NaHCO_3_, 0.1 M NaOH, and 1 M HCl. The remaining P content was determined as residual-P after digestion with H_2_SO_4_ and H_2_O_2_. The P fractions were categorized by bioavailability into three groups: labile P (resin-P and NaHCO_3_-P), moderately labile P (NaOH-P), and stable P (HCl-P and residual-P).

### 2.4 DNA extraction, sequencing, and gene quantification

DNA was extracted from a 0.5 g soil sample using the FastDNA Spin kit (MP Biomedicals, USA), and its concentration was measured with a Thermo NanoDrop spectrophotometer. The integrity of PCR products after amplification was assessed by electrophoresis on a 1.8% agarose gel. The V4-V5 region of bacterial 16S rRNA was amplified by PCR using the 341F/806R primer (5’-ACTCCTACGGGAGGCAGCA-3’ and 5’- GGACTACHVGGGTWTCTAAT-3’). The ITS1 region of fungi was amplified to PCR amplification using the ITS1F/ITS2R primers (5’-CTTGGTCATTTAGAGGAAGTAA-3’ and 5’-GCTGCGTTCTTCATCGATGC-3’). Library sequencing was performed on the Illumina NovaSeq 6000 platform. The raw data are available in the NCBI Sequence Read Archive (PRJNA1274922).

Raw reads were filtered to remove sequencing adapters, short reads (length < 50  bp) and low-quality reads to obtain high-quality clean reads. Paired-end reads were spliced using USEARCH v10 (Edgar, 2013), and then chimeras were identified and removed by UCHIME v4.2. The amplicon sequence variants (ASVs) present in each sample were inferred using USEARCH v10. Taxonomic assignment was carried out for the ASVs against Silva.138 (Quast et al., 2013) and UNITE (Kõljalg et al., 2005) as bacterial and fungal databases, respectively. Before further analysis, resampling was performed based on the minimum sequence numbers across all samples (73784 for bacteria and 64719 for fungi).

### 2.5 Statistical analysis

A one-way ANOVA with Duncan’s test was conducted using IBM SPSS Statistics to assess variance and significance among fertilization patterns at *P* < 0.05. R (v.4.1.0) was used to carry out the following analyses. The “vegan” package was adopted to conduct redundancy analysis (RDA) to determine the influence of soil properties on the variation in N and P fractions (Oksanen et al., 2019). Principal component analysis (PCA) to performed to evaluate the distribution of microorganisms, and permutational multivariate analysis of variance (PERMANOVA) was used to estimate the effect of fertilization pattern and soil position on soil microbial distribution. FEAST was employed to identify potential sources of rhizosphere microbiomes (Shenhav et al., 2019). The “psych” package was employed to analyze the correlation among ASVs (relative abundance > 0.05%) and construct a co-occurrence network (Revelle, 2021), and an absolute value of Spearman’s correlation coefficient |*r*| > 0.9 and *P* < 0.01 for the co-occurrence patterns were considered as statistically robust (Li et al., 2015a). The network diagram was visualized, and the topology parameters were calculated using Gephi 0.9.2 (Bastian et al., 2009). The Mantel test (using the “ggcor” package) was used to characterize the relationship between soil microbial communities and nutrient contents. Correlation analysis and random forest (RF) modeling analyses were conducted using the “psych” and “randomForest” packages to evaluate the relationships between microbial taxonomy and nutrient contents.

## 3 Results

### 3.1 Wheat yield and nutrient accumulation

**Figure 1:**
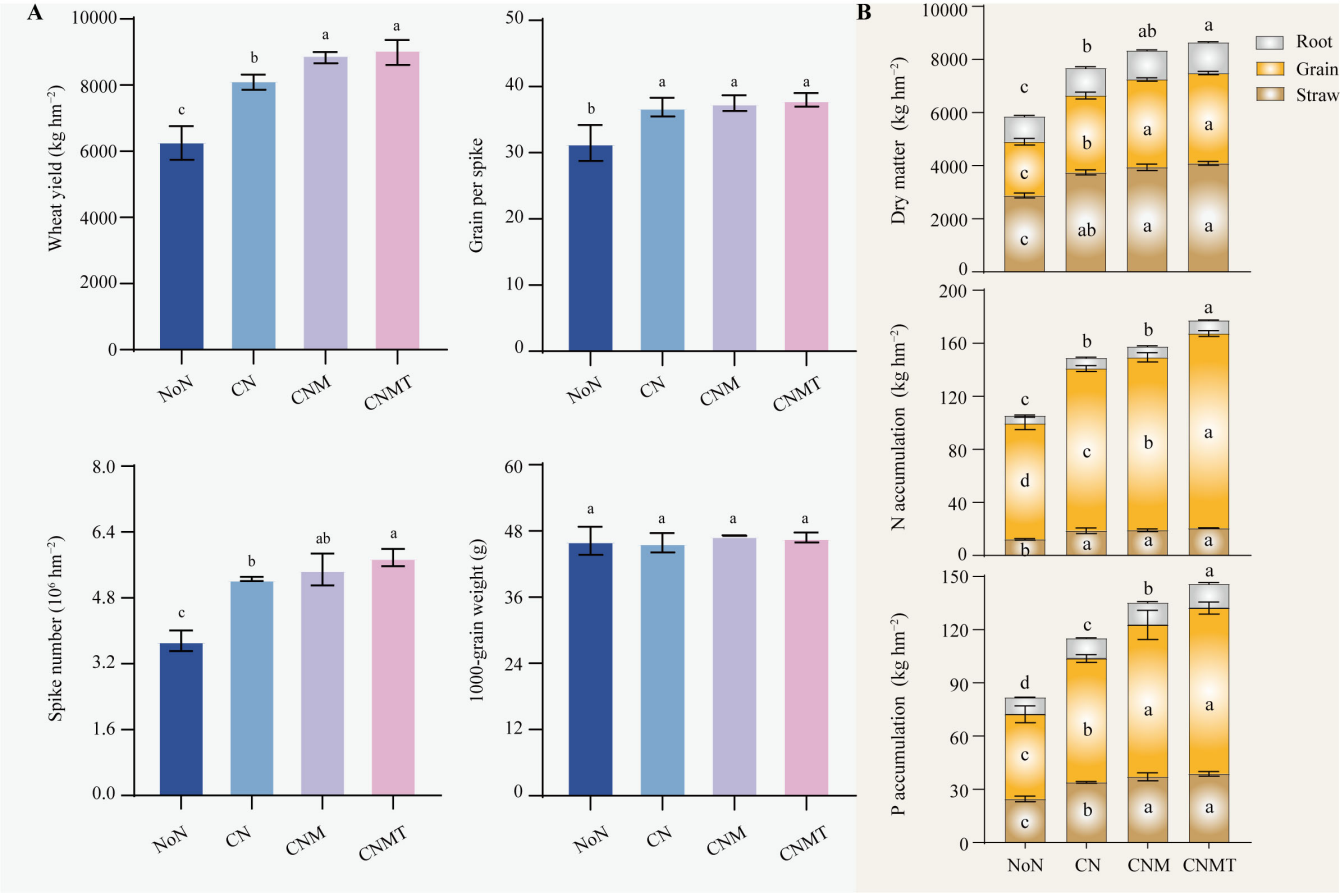
Wheat yield and its yield components **(A)**. Wheat dry matter, N, and P accumulation **(B)**. Error bars indicate the standard deviation (*n* = 3). Different letters represent significant (*P* < 0.05 level) differences among treatments. NoN, no N; CN, chemical fertilizer N; CNM, 80% chemical fertilizer N + 20% manure N; CNMT, 80% chemical fertilizer N + 20% manure N+ *Trichoderma*.

Compared with the NoN, N application (CN, CNM, and CNMT) significantly increased wheat yield, grains per spike, and spike number, with average increases of 38.6%, 19.1%, and 46.7%, respectively (**Figure 1A**). Among all treatments, CNMT produced the highest yield increase. Compared to CN, CNMT significantly increased wheat yield and spike number by 11.4% and 9.9%, respectively. Compared to NoN, N application significantly increased the dry matter weight in straw, grain, and roots, with average increases of 36.5%, 58.1%, and 45.4%, respectively (**Figure 1B**). Relative to CN, CNMT significantly enhanced dry matter accumulation in grains and roots, showing mean increases of 17.4% and 11.9%, respectively.

Compared to the NoN, N application significantly boosted N and P accumulation in plants (**Figure 1B**). Compared to CN, CNM and CNMT significantly increased N and P accumulation in grain, and P accumulation in straw and root. The accumulation of N and P in the roots was significantly enhanced by CNMT compared to CNM.

### 3.2 Soil physical and chemical properties

Compared with NoN (8.37 and 8.41), N application reduced rhizosphere and non-rhizosphere soil pH by 0.12−0.17 units and 0.12−0.14 units, respectively (**Table 1**). N application significantly increased the contents of soil organic matter (6.3%−18.5%), NH_4_^+^-N (8.7%−19.3%), NO_3_^−^-N (18.8%−32.4%), available P (14.2%−30.2%), and available K (2.9%−12.7%) in both rhizosphere and non-rhizosphere soils. Compared with CN, both CNM and CNMT significantly increased soil organic matter content in rhizosphere and non-rhizosphere soils, with average increases of 9.1% and 6.1%, respectively. CNMT significantly enhanced available P content in both rhizosphere and non-rhizosphere soils, showing an average increase of 6.7% compared with CN. Compared with the CNM, CNMT significantly increased the contents of NH_4_^+^-N, available P, and available K in rhizosphere soil, while significantly increased the contents of available P and K in non-rhizosphere soil.

**Table 1:** Basic properties in rhizosphere (R) and non-rhizosphere (NR) soil under different fertilization treatments.

Sampling position	Treatment	pH	Organic matter	NH_4_^+^-N	NO_3_^−^-N	Available P	Available K
			g kg^−1^	mg kg^−1^			
R	NoN	8.37 ± 0.03a	22.9 ± 0.2c	5.82 ± 0.2c	71.3 ± 1.1b	7.36 ± 0.2c	251 ± 4c
	CN	8.25 ± 0.05b	24.4 ± 0.4b	6.94 ± 0.3a	94.4 ± 4.4a	8.40 ± 0.2b	274 ± 6ab
	CNM	8.20 ± 0.05bc	26.0 ± 0.8a	6.32 ± 0.1b	91.4 ± 4.1a	8.73 ± 0.3b	266 ± 4b
	CNMT	8.24 ± 0.03b	27.2 ± 1.1a	6.83 ± 0.2a	93.9 ± 4.3a	9.58 ± 0.2a	279 ± 8a
NR	NoN	8.41 ± 0.02a	16.4 ± 0.3c	5.51 ± 0.2b	46.8 ± 1.8b	6.64 ± 0.2c	180 ± 6c
	CN	8.29 ± 0.02b	18.1 ± 0.2b	6.40 ± 0.2a	59.7 ± 3.6a	8.01 ± 0.2b	197 ± 8ab
	CNM	8.27 ± 0.03b	19.0 ± 0.4a	6.19 ± 0.2a	55.6 ± 3.1a	8.14 ± 0.2b	185 ± 5bc
	CNMT	8.28 ± 0.01b	19.3 ± 1.0a	6.32 ± 0.4a	57.7 ± 1.7a	8.60 ± 0.2a	203 ± 6a

NoN, no N; CN, chemical fertilizer N; CNM, 80% chemical fertilizer N + 20% manure N; CNMT, 80% chemical fertilizer N + 20% manure N+*Trichoderma*; SOM, organic matter; AP, available P; AK, available K. Values represent means ± standard error (*n* = 3). Different letters represent significant difference at *P* < 0.05.

### 3.3 Soil N fraction concentrations

Compared with NoN, N application significantly increased the contents of AHN and AAN in rhizosphere soil, with average increases of 8.4% and 5.6%, respectively (**Table 2**). Both CNM and CNMT significantly enhanced ASN content in rhizosphere soil, showing an average increase of 18.6% compared with NoN. N application significantly increased the contents of AHN, AHAN, AAN, and ASN in non-rhizosphere soil, with average increases of 6.4%, 7.2%, 5.8%, and 23.1%, respectively. Compared with CN, both CNM and CNMT significantly enhanced ASN content in rhizosphere and non-rhizosphere soils, showing increases of 15.4%−21.8% and 6.8%−12.2%, respectively. Compared with CNM, CNMT significantly increased AHAN content in rhizosphere soil, while significantly increased the contents of AHN and AAN in non-rhizosphere soils.

**Table 2:** Organic N fractions in rhizosphere (R) and non-rhizosphere (NR) soil under different fertilization treatments (mg kg^−1^).

Sampling position	Treatment	AHN	AHAN	AAN	ASN	AHUN
R	NoN	673 ± 2.8b	238 ± 0.7b	269 ± 4.4b	59.7 ± 1.2b	106 ± 7.6a
	CN	714 ± 2.4a	240 ± 1.2b	279 ± 5.5a	63.3 ± 1.2b	131 ± 2.5a
	CNM	728 ± 2.4a	243 ± 2.8b	281 ± 3.7a	73.1 ± 5.6a	132 ± 15a
	CNMT	745 ± 4.9a	255 ± 4.9a	284 ± 2.1a	77.1 ± 4.9a	130 ± 11a
NR	NoN	672 ± 6.5c	204 ± 5.5b	261 ± 3.2c	51.6 ± 4.6c	156 ± 12a
	CN	701 ± 3.7b	213 ± 5.5b	272 ± 2.5b	59.3 ± 1.2b	156 ± 14a
	CNM	718 ± 1.4b	220 ± 11.5ab	274 ± 0.7b	63.7 ± 1.9ab	159 ± 5.6a
	CNMT	727 ± 7.4a	224 ± 1.2a	280 ± 2.1a	67.0 ± 1.9a	156 ± 6.8a

NoN, no N; CN, chemical fertilizer N; CNM, 80% chemical fertilizer N + 20% manure N; CNMT, 80% chemical fertilizer N + 20% manure N+ *Trichoderma*; AHN, Acid hydrolysis nitrogen; AHAN, acid hydrolysis ammonium nitrogen; AAN, acid hydrolysis amino nitrogen; ASN, acid hydrolysis amino sugar nitrogen; AHUN, acid hydrolysis of unknown nitrogen. Values represent means ± standard error (*n* = 3). Different letters indicate significant difference at *P* < 0.05.

**Figure 2:**
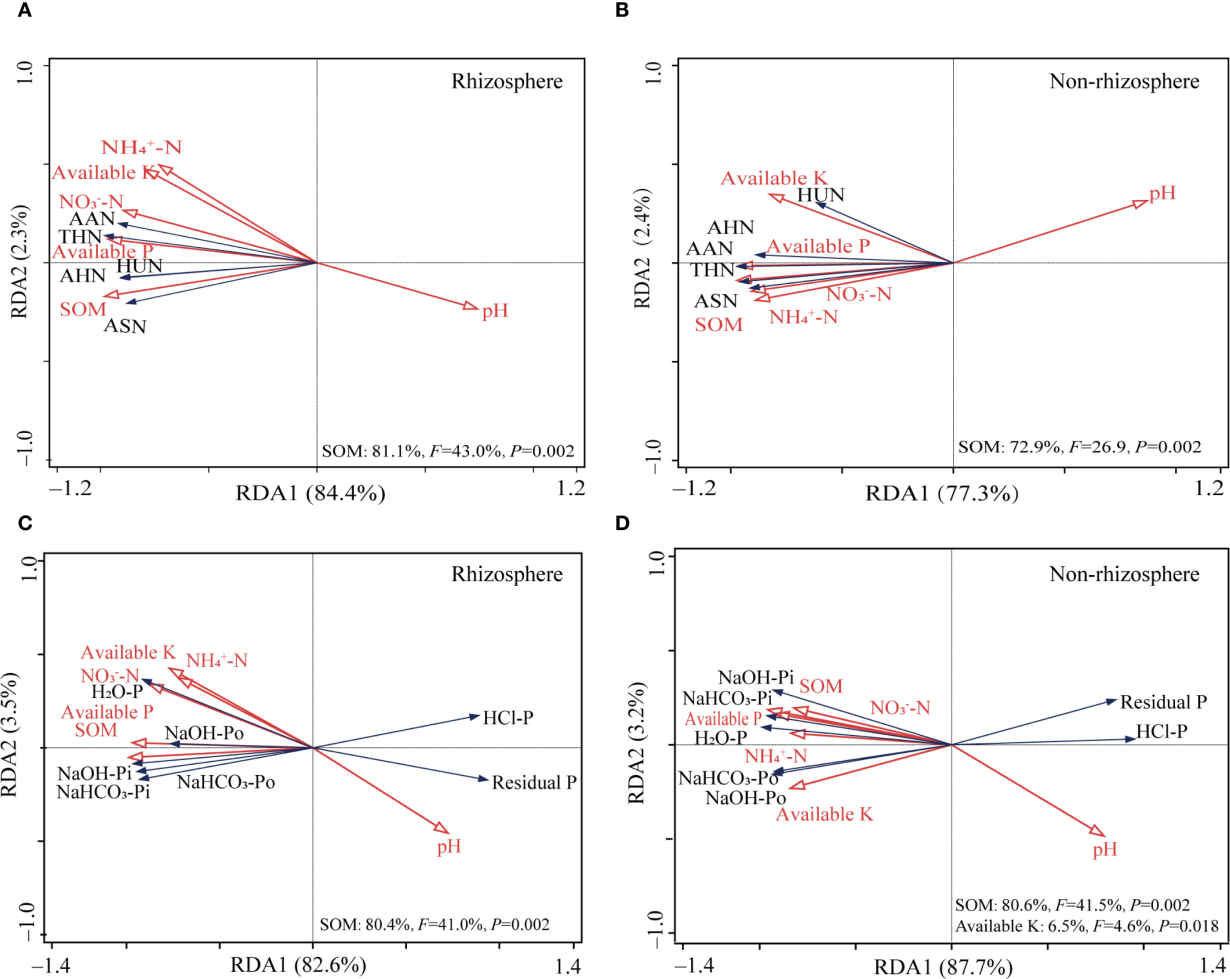
Redundancy analysis plots showing correlations between soil properties and soil N **(A, B)** and P fractions **(C, D)** in rhizosphere and non-rhizosphere soil. SOM, soil organic matter; AHN, Acid hydrolysis nitrogen; AHAN, acid hydrolysis ammonium nitrogen; AAN, acid hydrolysis amino nitrogen; ASN, acid hydrolysis amino sugar nitrogen; AHUN, acid hydrolysis of unknown nitrogen.

RDA results of rhizosphere soil physicochemical properties and organic N components revealed that the first principal component (RDA1) and second principal component (RDA2) accounted for 84.4% and 2.3% of the total variance, respectively (**Figure 2A**). Soil organic matter (*F* = 43.0, *P* = 0.002) significantly influenced the distribution of N components in rhizosphere soil, explaining 81.1% of the total variance. In non-rhizosphere soil (**Figure 2B**), RDA1 and RDA2 accounted for 77.3% and 2.4% of the total variance, respectively. Soil organic matter significantly influenced the distribution of N components (*F* = 26.9, *P* = 0.002), explaining 72.9% of the total variance.

### 3.4 Soil P fraction concentrations

In rhizosphere soil, compared with NoN, N application resulted in a significant average increase of 43.0% in H_2_O-P content, while it significantly decreased dil. HCl-P and residual-P contents by 10.1% and 33.6%, respectively (**Table 3**). Compared with CN, both CNM and CNMT significantly elevated NaHCO_3_-Pi and NaOH-Pi contents, with average increases of 48.6% and 23.3%, respectively. Compared with CN, CNMT significantly increased NaHCO_3_-Pi, NaHCO_3_-Po, NaOH-Pi, NaOH-Po contents. In non-rhizosphere soil, N application significantly increased H_2_O-P, NaHCO_3_-Pi, and NaOH-Pi contents by 65.3%, 123%, and 28.4% on average, respectively, while CNM and CNMT significantly increased NaHCO_3_-Po and NaOH-Po contents, with average increases of 154% and 15.4%, respectively. Compared with CN, CNM and CNMT significantly enhanced H_2_O-P, NaHCO_3_-Pi, NaHCO_3_-Po, NaOH-Pi, and NaOH-Po contents in non-rhizosphere soil, exhibiting average increases of 35.5%, 51.0%, 122%, 12.8%, and 20.1%, respectively. Compared with the CNM, CNMT significantly increased the contents of NaHCO_3_-Pi and NaOH-Pi in rhizosphere soil, while significantly increased the contents of H_2_O-P, NaHCO_3_-Pi, and NaHCO_3_-Po.

**Table 3:** Chemical P fractions in rhizosphere (R) and non-rhizosphere (NR) soil under different fertilization treatments (mg kg^−1^).

Sampling position	Treatment	H_2_O-P	NaHCO_3_-Pi	NaHCO_3_-Po	NaOH-Pi	NaOH-Po	dil.HCl-P	Residual-P
		Labile P			Moderately labile P		Stable P	
R	NON	2.63 ± 0.1b	11.4 ± 1.6d	13.3 ± 0.3c	14.9 ± 0.4c	20.6 ± 0.9b	252 ± 6.7a	29.6 ± 2.5a
	CN	3.61 ± 0.2a	14.2 ± 0.8c	14.9 ± 1.0bc	16.3 ± 0.5c	22.3 ± 1.1b	229 ± 7.1b	22.3 ± 1.5b
	CNM	3.75 ± 0.1a	19.6 ± 0.9b	16.5 ± 1.3ab	19.2 ± 1.3b	23.3 ± 2.8ab	228 ± 2.1b	18.6 ± 1.0b
	CNMT	3.92 ± 0.2a	22.6 ± 1.7a	17.5 ± 0.9a	21.0 ± 0.7a	25.9 ± 1.4a	223 ± 4.0b	18.0 ± 0.5c
NR	NON	4.54 ± 0.1d	11.5 ± 1.5d	6.23 ± 1.7c	17.5 ± 1.1c	25.4 ± 1.6b	237 ± 4.5a	134 ± 2.2a
	CN	6.07 ± 0.2c	19.1 ± 1.0c	7.13 ± 0.4c	20.7 ± 0.5b	24.4 ± 1.0b	234 ± 19.6a	130 ± 8.7a
	CNM	7.77 ± 0.2b	27.4 ± 0.9b	13.4 ± 0.9b	23.0 ± 1.5a	28.4 ± 1.0a	227 ± 20.3a	126 ± 8.9a
	CNMT	8.68 ± 0.4a	30.3 ± 1.5a	18.2 ± 1.5a	23.7 ± 0.6a	30.2 ± 1.3a	217 ± 2.3a	121 ± 2.6a

NoN, no N; CN, chemical fertilizer N; CNM, 80% chemical fertilizer N + 20% manure N; CNMT, 80% chemical fertilizer N + 20% manure N+Trichoderma. Values represent means ± standard error (*n* = 3). Different letters indicate significant difference at *P* < 0.05.

RDA of physicochemical properties and P fractions revealed that RDA1 and RDA2 accounted for 82.6% and 3.5% of the total variance, respectively (**Figure 2C**). Soil organic matter significantly influenced the distribution of P fractions (*F* = 41.0, *P* = 0.002), explaining 80.4% of the total variance. In non-rhizosphere soil, RDA1 and RDA2 accounted for 87.7% and 3.2% of the total variance, respectively (**Figure 2D**). Both soil organic matter (*F* = 41.5, *P* = 0.002) and available K (*F* = 4.6, *P* = 0.018) significantly influenced the distribution of P fractions, explaining 80.6% and 6.5% of the total variance, respectively.

### 3.5 Soil microbial community structure and function

**Figure 3:**
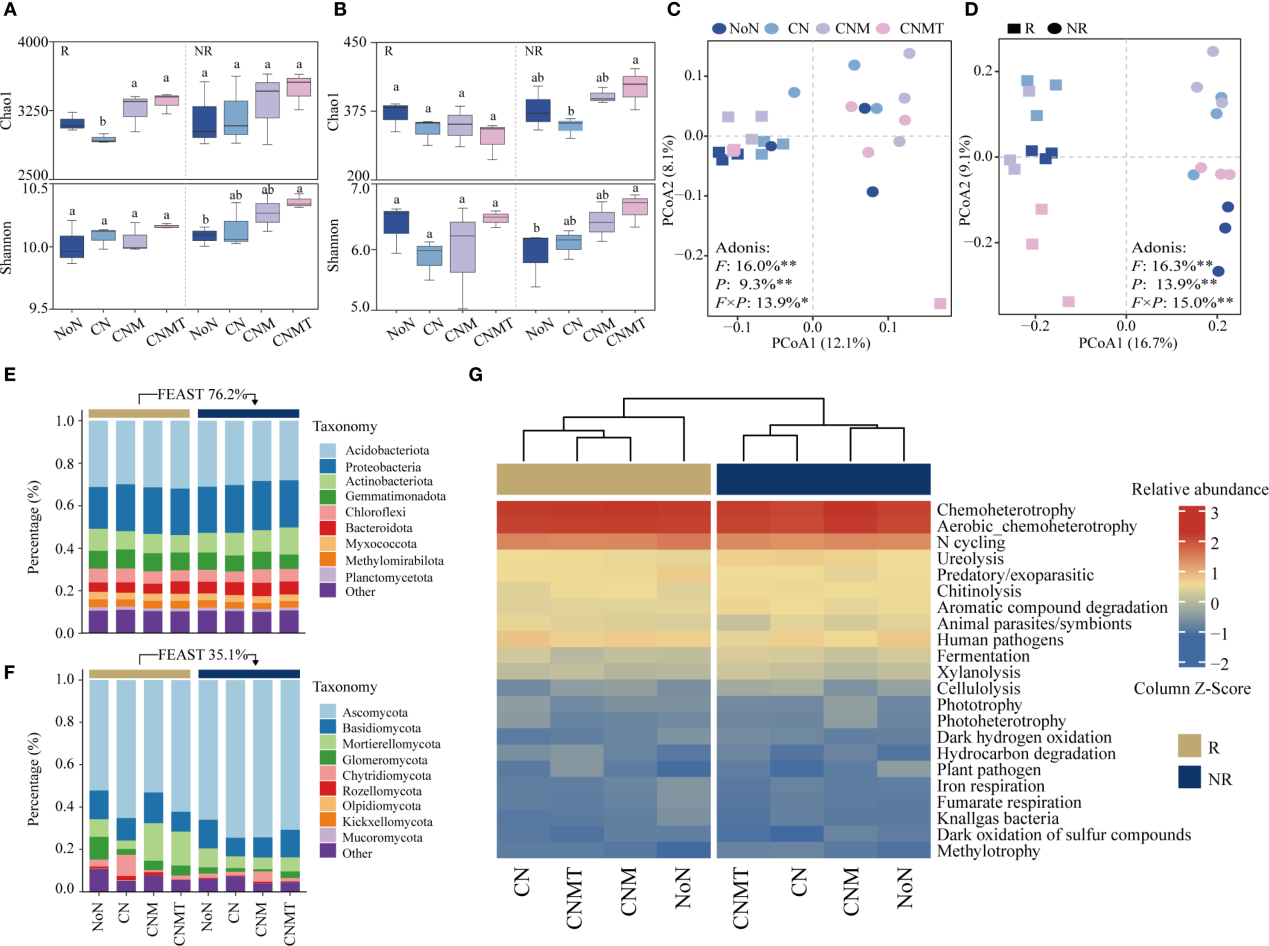
The differences in α-diversity (Chao1 and Shannon index) of the bacterial **(A)** and fungal communities **(B)**. Principal coordinate analysis ordinations based on the Bray-Curtis dissimilarity matrices showing changes in bacterial **(C)** and fungal communities **(D)** (F, fertilization treatment; P, soil position). Adonis analysis showing the effects of the fertilization treatment and soil position on the microbial communities (**P < 0.01; *P < 0.05). Changes in the relative abundances of bacterial **(E)** and fungal **(F)** taxonomic (at the phylum level). Heatmap of metabolic and ecological functions of bacteria based on FAPROTAX prediction **(G)**.

In rhizosphere soil, compared with CN, both CNM and CNMT significantly increased bacterial α-diversity (Chao1 index) by 10.8% and 13.8%, respectively (**Figure 3A**). In non-rhizosphere soil, compared with CN, CNMT significantly increased the fungal Chao1 index by 24.0% (**Figure 3B**). Compared to NoN, CNMT significantly increased both the bacterial and fungal Shannon indices in non-rhizosphere soil. The PCoA results showed a clear separation of the microbial communities in rhizosphere and non-rhizosphere soils (**Figures 3C, D**). PERMANOVA analysis indicated that 39.2% of the variations observed in the bacterial community could be attributed to fertilization patterns (16.0%), soil position (9.3%), and the interactive effect of these two factors (13.9%). The fertilization patterns (16.3%), soil position (13.9%), and the interactive effect (15.0%) of these two factors significantly altered the composition of fungal communities.

The FEAST results showed that rhizosphere bacterial communities were derived from non-rhizosphere soil (76.2%) (**Figure 3E**). A phylum-based taxonomic classification showed that Acidobacteriota (accounting for 28.0%–31.9%), Proteobacteria (19.6%–23.2%), and Actinobacteriota (6.8%–8.8%) were the main taxa in both rhizosphere and non-rhizosphere soil. In rhizosphere soil, N application increased the relative abundance of Proteobacteria and Gemmatimonadota. Compare to CN, both CNM and CNMT increased the relative abundance of Acidobacteriota. In non-rhizosphere soil, compare to CN, both CNM and CNMT decreased the relative abundance of Acidobacteriota, while increasing the relative abundance of Gemmatimonadota and Chloroflexi. The FEAST results showed that rhizosphere fungal communities were derived from non-rhizosphere soil (35.1%) (**Figure 3F**). The fungal community was dominated by members of Ascomycota (accounting for 52.1%–74.4%), Basidiomycota (8.7%–14.5%), and Mortierellomycota (4.0%–17.7%). In rhizosphere soil, compared with CN, both CNM and CNMT decreased the relative abundance of Ascomycota and Chytridiomycota, while increasing the relative abundance of Mortierellomycota and Glomeronmycota.

**Figure 4:**
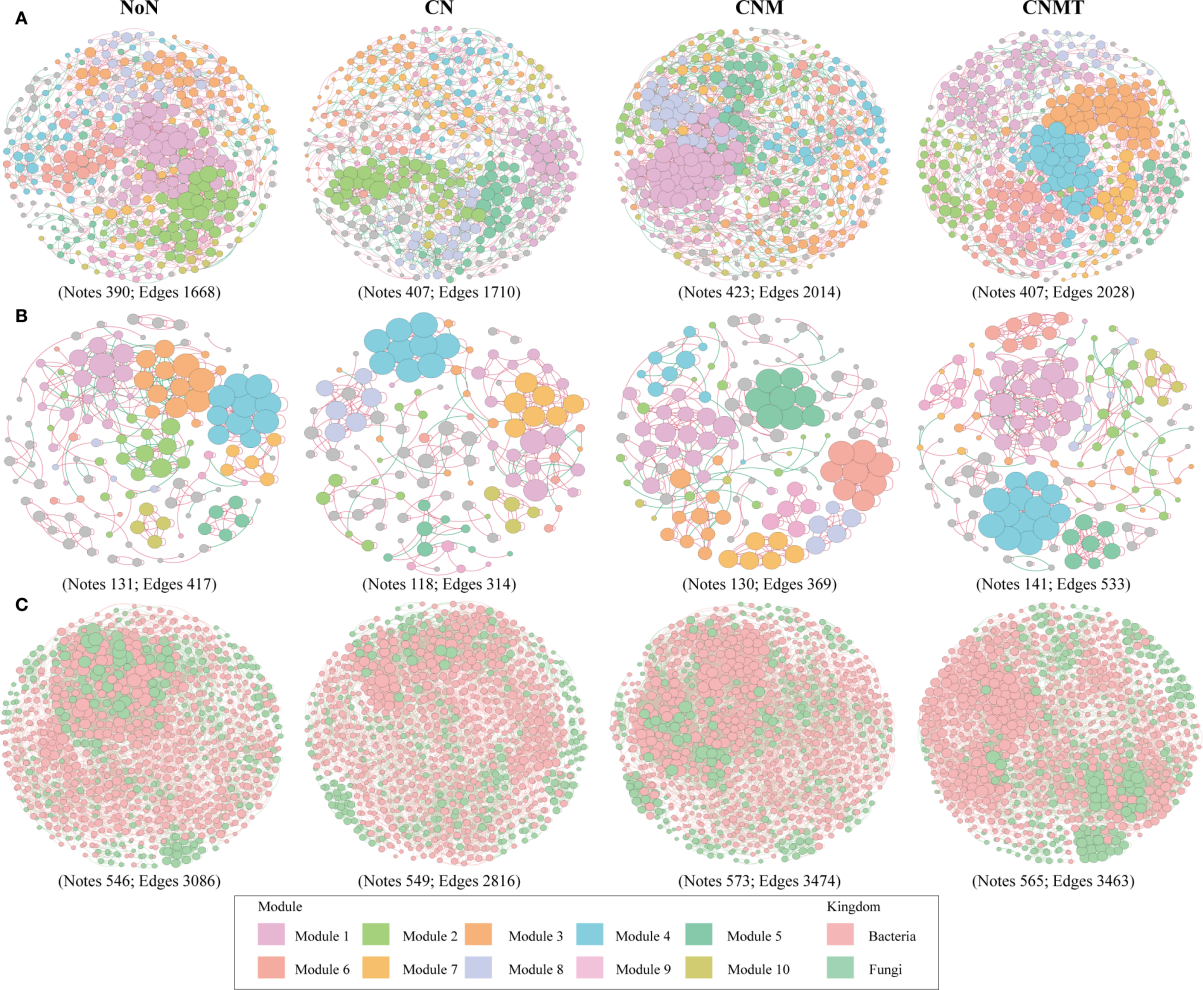
Ecological network analysis of bacterial **(A)**, fungal **(B)**, and bacterial-fungal **(C)** community networks in fertilization treatment. Node represents individual ASV (Spearman correlations, *P* < 0.01 and *r* > 0.9).

The functional annotation of prokaryotic taxa (FAPROTAX) analysis showed that the main potential functions were related to chemoheterotrophy and N cycling (**Figure 3G**). Compared to CN, N cycling and chitinolysis were overrepresented in CNMT in both rhizosphere and non-rhizosphere soil. Microbial interaction (bacteria-bacteria, fungi-fungi, and bacteria-fungi) networks were constructed to visualize microbial co-occurrence patterns under different fertilization regimes (**Figure 4**). According to the topological parameters of each network (**Table 4**), the bacteria-bacteria networks in CNM and CNMT exhibited higher complexity than those in NoN and CN, based on the higher note number, edge number, and average degree. The fungi-fungi network in CNMT showed higher complexity, with a higher note number, edge number, average degree, and graph density. The bacteria-fungi networks in CNM and CNMT also showed higher complexity, with a higher note number, edge number, average degree, and graph density.

**Table 4:** Basic topological parameters for occurrence networks within different aggregates.

Topological parameters		Fertilization treatment			
		NoN	CN	CNM	CNMT
Bacteria-Bacteria	Nodes	390	407	423	407
	Edges	1668	1710	2014	2028
	Average degree	4.277	4.201	4.761	4.983
	Graph density	0.011	0.010	0.011	0.012
	Modularity	0.692	0.746	0.701	0.684
	Average clustering coefficient	0.228	0.269	0.250	0.237
	Average path length	7.690	7.761	7.820	8.083
Fungi-Fungi	Nodes	131	118	130	141
	Edges	417	314	369	533
	Average degree	3.183	2.661	2.838	3.780
	Graph density	0.024	0.023	0.022	0.027
	Modularity	0.732	0.879	0.850	0.792
	Average clustering coefficient	0.411	0.416	0.380	0.394
	Average path length	2.331	1.389	1.488	1.859
Bacteria-Fungi	Nodes	546	549	573	565
	Edges	3086	2816	3474	3463
	Average degree	5.562	5.129	6.063	6.129
	Graph density	0.010	0.009	0.011	0.011
	Modularity	0.688	0.753	0.712	0.719
	Average clustering coefficient	0.278	0.304	0.297	0.286
	Average path length	8.135	7.718	7.471	10.31

### 3.6 Relationship between soil nutrients and microorganisms

**Figure 5:**
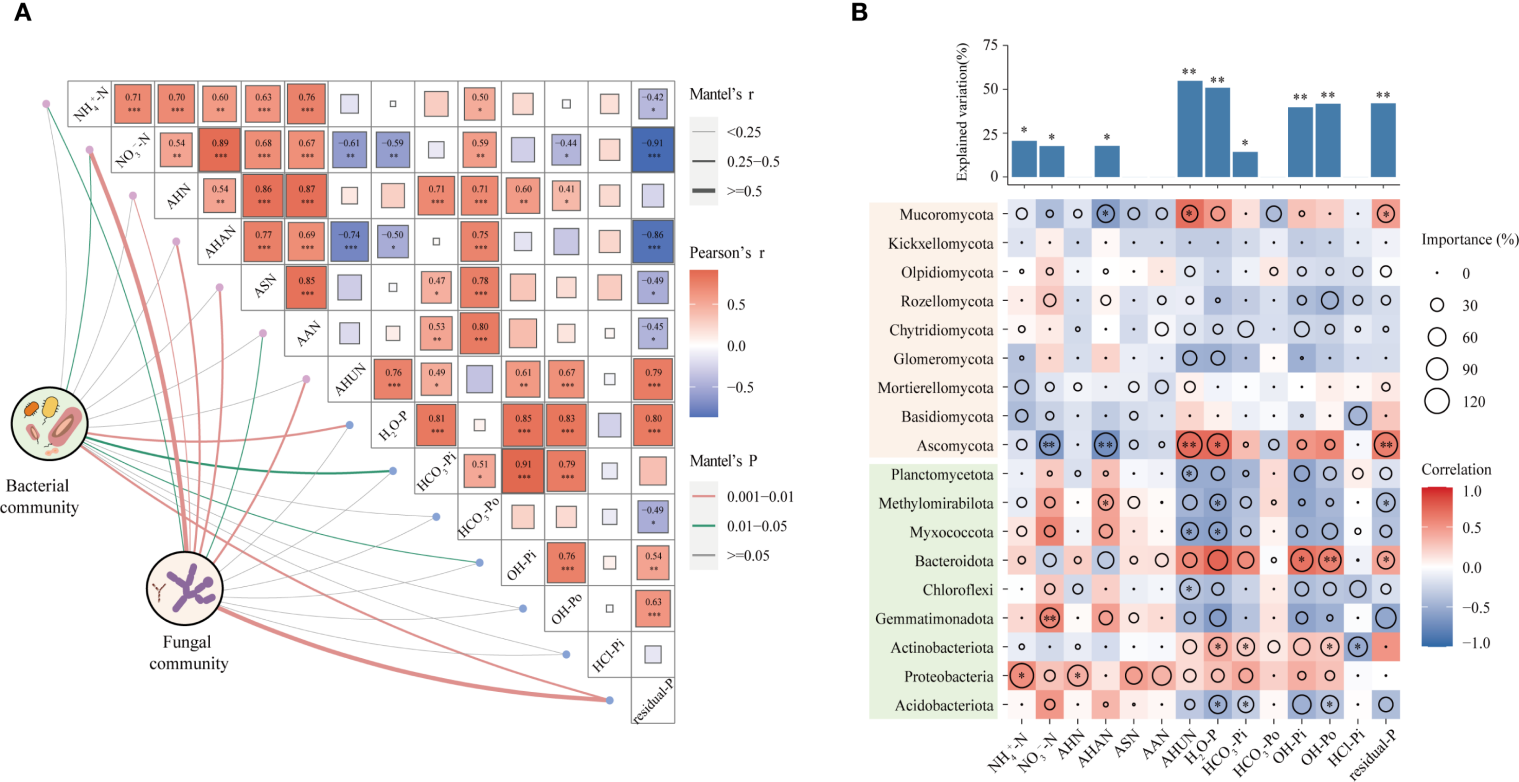
Relationships between the microbial community composition and soil N and P fractions **(A)**. Associations between soil organic N and P fractions and the relative abundances of bacterial and fungal taxa (at the phylum level) **(B)**. Circle size represents the importance (%IncMSE) of predictors explaining the variation in microbial abundance based on the random forest modeling. The “rfPermute” function was employed to conduct permutation tests and assess the *P*-values for variable importance. The height of the columns in the above bar chart represents the degree of interpretation of soil nutrient variables. The significance was calculated using the A3 package. *P < 0.05; **P < 0.01. Colors represent Spearman correlations.

The Mantel test showed that NO_3_^−^-N, H_2_O-P, NaHCO_3_-Pi, NaOH-Pi, and residual-P were significantly related to the bacterial community composition (*r* > 0.25; *P* < 0.05; **Figure 5A**), while NH_4_^+^-N, NO_3_^−^-N, AHN, AHAN, ASN, AAN, AHUN, and residual-P showed significant relationships with the fungi community (*r* > 0.25; *P* < 0.05). The relevant analysis results show that NH_4_^+^-N and NO_3_^−^-N were positively correlated to AHN, AHAN, ASN, AAN, and NaHCO_3_-Po. H_2_O-P was positively correlated with NaHCO_3_-Pi, NaOH-Pi, NaOH-Po, and residual-P.

Spearman correlation showed that NO_3_^−^-N and AHAN were positively correlated with Acidobacteriota, Proteobacteria, Gemmatimonadota, Mycococcota, and Methlomirabilota, while they were negatively correlated to Ascomycota and Mucormycota (**Figure 5B**). Proteobacteria had a positive relationship with N and P fractions. H_2_O-P, NaHCO_3_-Pi, NaOH-Pi, NaOH-Po were negatively correlated with Acidobacteriota, Gemmatimonadota, Chloroflexi, Mycococcota, and Methlomirabilota, while they were positively correlated with Bacteroidota and Ascomycota. The RF analysis indicated that specific bacterial and fungal taxa were pivotal in explaining the observed variations among N and P fractions. The NH_4_^+^-N (20.6%, *P* < 0.05), NO_3_^−^-N (17.6%, *P* < 0.05), AHAN (17.7%, *P* < 0.05), AHUN (54.8%, *P* < 0.01), H_2_O-P (50.8%, *P* < 0.01), NaOH-Pi (39.7%, *P* < 0.01), NaOH-Po (41.7%, *P* < 0.01), and residual-P (41.9%, *P* < 0.01) were significantly affected by bacterial and fungal taxa.

## 4 Discussion

### 4.1 Combined application of manure and *Trichoderma* increased the availability of N and P

Organic N constitutes the primary form of N present in soil (≥ 90%), functioning as both a source and a reservoir for mineral N. The content and compositional status of organic N critically influence the availability of N within the soil (Stevenson, 1982). Wu et al. (2021) demonstrated that the practice of returning straw to the field led to increases in the concentrations of AHAN, ASN, and AHUN. A recent study found that replacing inorganic fertilizers with organic options, particularly a mix of corn straw and pig manure, enhanced various AHN fractions, thereby boosting soil N sequestration and supply, which ultimately improved greenhouse vegetable yields (Yuan et al., 2024). Further, this study found that the synergistic effect of manure and *Trichoderma* (CNMT) significantly enhanced soil AHAN and ASN contents in both rhizosphere and non-rhizosphere soils (**Table 2**). ASN, a key component of the microbial cell wall, includes constituents such as glucosamine and muramic acid, which are primarily derived from soil microorganisms (Roberts et al., 2007). AHAN serves as an N source for plants and microbes, originating from NH_4_^+^-N and the breakdown of various compounds (Ren et al., 2021). Both AHAN and ASN are considered active organic N fractions, linked to microbial activity and crop absorption, indicating the soil’s N supply potential (Mishra et al., 2005). Our study demonstrated that CNMT increased the contents of AHAN and ASN in both rhizosphere and non-rhizosphere soils. This effect can be attributed to several factors: (1) the inherent presence of a substantial number of microorganisms within the organic materials themselves; (2) the provision of C sources (energy) by the applied organic fertilizer, which facilitates the proliferation of soil microorganisms; and (3) the well-documented ability of Trichoderma to secrete chitinases and proteases is hypothesized to be instrumental in the mineralization of organic N compounds (Mallikharjuna Rao et al., 2016; Kappel et al., 2020). Consistent with this potential mechanism, we observed that microbial N cycling and chitinolytic functional potential were high under CNMT in both rhizosphere and non-rhizosphere soils (**Figure 3G**). While this correlation supports the inference, future studies directly measuring enzyme activities are needed to establish causation. Simultaneously, CNMT enhanced wheat N accumulation (**Figure 1B**), suggesting an improvement in plant N uptake efficiency. This enhancement can be attributed to the substantial potential of soil N supply. Moreover, this study found that soil organic matter significantly influenced the distribution of N components in rhizosphere soil, which supports the results by Wu et al. (2019). The primary reason is that the potential transformation of soil organic N may be constrained by the availability of C sources. Under conditions where soil organic matter is limited, microorganisms may utilize low-molecular-weight nitrogenous compounds, such as amino acids and amino sugars, as alternative C sources (Su et al., 2022).

Many studies have shown that manure application increases labile P content in soils with various crops, which is beneficial to plant uptake (Liu et al., 2020; Zhang et al., 2024b). Similarly, in this study, CNMT significantly increased the contents of labile P (NaHCO_3_-Pi and NaHCO_3_-Po) and moderately labile P (NaOH-Pi and NaOH-Po) in both rhizosphere and non-rhizosphere soils (**Table 3**). Further, our study demonstrated that the combined application of manure and *Trichoderma* significantly enhances the availability of P in the soil. This effect may be attributed to *Trichoderma’s* capability to solubilize inorganic P through the secretion of organic acids and to mineralize organic P via phosphatase activity (Bononi et al., 2020; Duan et al., 2023). However, since extracellular enzyme activities were not quantified in this study, the contribution of these specific mechanisms requires further validation. Moreover, the elevated content of labile P indicates an increased P nutrient supply for crop, consequently leading to enhanced wheat P accumulation in CNMT (**Figure 1B**). Notably, soil organic matter was the primary driver of P fraction distribution. This suggests that: (1) manure-enhanced organic matter underpins *Trichoderma’s* nutrient-mobilizing efficacy; 2) increasing soil organic matter affects P transformation, for example, organic acids decrease the formation of more stable crystalline Ca phosphate, promote soil aggregate stability, and reduce the downward migration of available P; and enhancing microbial activity can promote the activation of P (Zhang et al., 2023; Krause et al., 2025). The residual-P in non-rhizosphere soil (121−134 mg kg^−1^) are 4−7 times higher than rhizosphere (18−29 mg kg^−1^), as a result of rhizosphere priming effects and microbial mobilization processes. This phenomenon is likely driven by: 1) Plant roots releasing protons and organic acids to solubilize inorganic P, enhancing root-zone chemical weathering and reducing residual P (Zheng et al., 2025). 2) Driven by root exudates, rhizosphere microbial activity is enhanced, converting P from stable forms to labile forms (Huang et al., 2021). 3) Plant roots rapidly absorb mobilized P, maintaining a steep diffusion gradient and depleting rhizosphere P pools, promoting conversion of residual P.

### 4.2 Combined application of organic fertilizer and *Trichoderma* enhanced microbial interactions

Microbial diversity is vital for soil quality, sustainability, and functionality, with high diversity indicating better soil health and plant productivity (Chen et al., 2020a; Wu et al., 2023). A growing body of evidence indicates that the incorporation of organic fertilizers into agricultural production systems enhances the diversity of soil microbial communities. For example, Francioli et al. (2016) demonstrated through a long-term fertilization experiment spanning 113 years that the application of manure significantly increases bacterial community diversity. Similarly, Yi et al. (2021) reported that in an 18-year peanut cropping system, manure application enhances bacterial diversity while having a neutral impact on fungal diversity. In this study, CNMT increased bacterial α-diversity (Chao1 index) in rhizosphere soil (**Figure 3A**), while it increased fungal α-diversity in non-rhizosphere soils (**Figure 3B**). These results could be attributed to the following: 1) organic fertilizers offer a richer supply of organic C and a wider range of nutrients compared to chemical fertilizers. They enhance soil structure and create a favorable environment for microorganisms by supplying energy and nutrients (Chen et al., 2022a; Zhang et al., 2022). 2) *Trichoderma* application alters metabolic processes, creates new ecological niches, affects root metabolite exudation, and competes for resources with native soil microorganisms, thereby stimulating or suppressing specific microbial taxa and causing changes in the soil microbiome and ecosystem function (Lucini et al., 2019; Wang et al., 2023). Moreover, the PCoA and PERMANOVA analyses revealed that fertilization patterns, sample position, and their interaction accounted for 40%–50% of the variation in the microbial community. The unexplained variation could be due to complex ecological processes not captured by PCoA (uses linear models Bray-Curtis), or the omission of key environmental factors such as specific nutrients, pH changes, and plant physiological states. For instance, Mantel results showed a significant correlation between the microbial community and N and P content (**Figure 5A**). This study showed that rhizosphere bacteria are primarily originate from the non-rhizosphere soil’s microbial seed bank, forming a subset of soil microbial communities. This is because the initial soil microbiome is shaped by soil conditions such as soil types and fertilization (Cheng et al., 2020; Zhang et al., 2024a). In addition, only 35% of rhizosphere fungi are derived from the non-rhizosphere soil’s microbial community; this is because the addition of exogenous fungi (*Trichoderma*) has a significant impact on soil fungi and shapes both the soil fungal community and the host plant (Hu et al., 2018).

Soil microorganisms do not exist in isolation; rather, they form intricate and interactive ecological networks. Investigating microbial co-occurrence networks is essential for elucidating microbe-microbe interactions and understanding ecosystem functioning (Cardona et al., 2016; Chen et al., 2022b). This study suggested that CNMT enhanced the complexity of microbial co-occurrence networks: bacteria-fungi networks exhibited higher node and edge counts as well as greater graph density, indicating intensified cross-kingdom collaborations, which support a higher diversity of interactions and greater functional complementarity (Peng et al., 2024). This could possibly be because *Trichoderma* likely amplified this synergy by secreting auxins that stimulate root exudation, thereby enriching C substrates for rhizosphere microbes (De Palma et al., 2019). This facilitated a “microbial hub” effect, in which Proteobacteria (positively correlated with N and P fractions; **Figure 5B**) mediated nutrient transformations. Consequently, microbial interactions under CNMT optimized nutrient flux, corroborating increased N and P accumulation in plants (**Figure 1B**). This indicates a significant relationship between microbial networks and soil functionality, with specific enrichment of Acidobacteriota (N-cycling specialists) and Proteobacteria (versatile nutrient scavengers). FAPROTAX analysis revealed heightened N-cycling and chitinolysis functions (**Figure 3G**), consistent with elevated ASN and NO_3_^-^-N levels. Overall, CNMT can reshape soil microbial communities, fostering complex interactions that facilitate nutrient cycling.

### 4.3 Combined application of organic fertilizer and *Trichoderma* improved wheat yield

In this study, CNMT improved wheat yield by increasing spike number (**Figure 1**). This can be attributed to the synergistic interaction between manure and *Trichoderma*: 1)The manure provides essential nutrients, creating optimal conditions for *Trichoderma*, which aids in decomposing manure and releasing nutrients. 2) This interaction enhances root growth, improving nutrient uptake and aboveground plant development (Harman, 2006; Salem et al., 2024). The study confirmed that CNMT increases root dry matter and nutrient accumulation, supporting previous findings. 3) The enhanced availability of N and P in the rhizosphere and non-rhizosphere soil by CNMT provides nutrients for wheat growth. Manure application added exogenous C to the soil, promoting the activation of microorganisms. The enhanced microbial activity, evidenced by the predicted increase in enzymatic functional potential (**Figure 3G**), is a primary driver behind the accelerated mineralization of organic nutrients (Zhang et al., 2017). 4) CNMT enhances microbial diversity and interactions, improving soil structure and nutrient transformation, which stabilizes yields. Enriched Acidobacteriota and Proteobacteria improve N mineralization and P mobility, directly supporting grain filling with a 17.4% increase in grain dry matter. Overall, applying manure and *Trichoderma* enhances soil microbial activity and nutrient availability for high wheat yield. Furthermore, soil properties (like texture, pH, and organic matter) and climate (temperature and precipitation) significantly affect nutrient transformation and microbial communities, with crop traits further influencing these interactions. This study focused on a field with lime concretion black soil, characterized by high calcium carbonate, moderate clay texture, and alkaline pH, under a wheat cropping system. Wheat’s root structure and exudates likely enhance specific microbial activities and nutrient release in this soil. Future research should adopt a multi-site, multi-soil-type approach, including black, red, and fluvo-aquic soils, across various climates to better understand the interactions between soil type, climate, and wheat traits.

## 5 Conclusion

The combined application of chemical fertilizers with organic manure and *Trichoderma* significantly increased wheat grain yield by enhancing N and P accumulation in plants and roots, as well as by increasing spike number. CNMT elevated the content of acid-hydrolyzable amino sugar N in both rhizosphere and non-rhizosphere soils, thereby enhancing the N supply potential. Simultaneously, it significantly increased labile and moderately labile P fractions, thereby improving soil P nutritional status and availability. Furthermore, it enhanced microbial community diversity and improved connectivity and stability of the microbial network. Overall, the combined application of chemical fertilizers, organic manure, and *Trichoderma* (80% chemical N + 20% organic N + Trichoderma) effectively improved N supply, P activation, and microbial regulation, boosting nutrient absorption and yield.

## Data Availability

The datasets presented in this study can be found in online repositories. The names of the repository/repositories and accession number(s) can be found below: https://www.ncbi.nlm.nih.gov/, PRJNA1274922.
